# Perceived awareness, objective nutrition knowledge, and health beliefs about probiotics and prebiotics among adults in the United Arab Emirates: a cross-sectional study

**DOI:** 10.3389/fnut.2026.1929193

**Published:** 2026-09-07

**Authors:** Nadia Alkalbani, Tareq M. Osaili, Reyad S. Obaid, Hayder Hasan, Leila Cheikh Ismail, Sakina Karu, Asala Nasser, Aliya Abdul Hameed, Ethar Mohamed, Musaina Nasrin, Tasneem Akbiyik

**Affiliations:** 1 Department of Clinical Nutrition and Dietetics, College of Health Sciences, University of Sharjah, Sharjah, United Arab Emirates; 2 Research Institute of Medical and Health Sciences, University of Sharjah, Sharjah, United Arab Emirates; 3 Department of Nutrition and Food Technology, Faculty of Agriculture, Jordan University of Science and Technology, Irbid, Jordan; 4 Nuffield Department of Women's & Reproductive Health, University of Oxford, Oxford, United Kingdom

**Keywords:** health beliefs, information sources, objective nutrition knowledge, perceived awareness, prebiotics, probiotics

## Abstract

**Background:**

Probiotics and prebiotics are increasingly promoted as functional food components. This growing visibility has not been matched by consistent public understanding of their definitions, dietary sources, and scientifically supported health claims. Against this background, this study assessed perceived awareness, objective knowledge, information sources, and health beliefs related to probiotics and prebiotics among adults in the United Arab Emirates (UAE). It also examined factors associated with adequate objective knowledge.

**Methods:**

A cross-sectional online survey was conducted among 744 adult UAE residents between April and May 2026. Perceived awareness was measured using a 10-point scale. Objective knowledge was assessed using four items on definitions and natural dietary sources, and adequate knowledge was defined as at least three correct answers. In addition, information sources and health beliefs were assessed, and multivariable logistic regression was used to identify factors associated with adequate objective knowledge.

**Results:**

Mean perceived awareness was 4.6 ± 2.8, and 18.0% of participants reported high awareness. Mean objective knowledge was 1.9 ± 1.6, with 39.9% demonstrating adequate knowledge. Correct responses were highest for the probiotic definition (55.8%) and lowest for natural prebiotic sources (37.0%). High perceived awareness and high objective knowledge overlapped in only 10.1% of participants. In the multivariable model, internet/websites (90.3%) and social media (84.9%) were the most frequently reported information sources, but neither was independently associated with adequate knowledge. By contrast, receiving information from healthcare professionals was positively associated with adequate knowledge [adjusted odds ratio (aOR) 1.83, *p* = 0.009]. Health beliefs were strongest for gut health and immunity and less certain for detoxification, obesity, stress, and mental health.

**Conclusion:**

Perceived awareness did not consistently correspond to objective knowledge. These findings indicate a nutrition-related knowledge gap, particularly for prebiotics and their natural dietary sources. Digital nutrition communication should be strengthened by professional guidance and clearer messages that distinguish probiotic and prebiotic concepts, dietary sources, and scientifically supported health claims.

## 1 Introduction

Probiotics and prebiotics are now common terms in food, supplement, and wellness communication, yet both concepts have precise scientific meanings. Probiotics refer to live microorganisms that provide health benefits when consumed in adequate amounts, whereas prebiotics are substrates selectively utilized by host microorganisms to produce a health benefit (1, 2). These definitions are important for public understanding because they clarify how food examples and product claims should be interpreted. For example, fermented foods are not automatically probiotic foods, and not all dietary fibers are prebiotics (3, 4). As microbiome-related products expand across foods, dietary supplements, and live biotherapeutic products, the public is increasingly exposed to terms that require more than surface familiarity (5–7).

This creates a nutrition communication problem because terms that have specific scientific requirements may be received by the public as general health claims (8). Messages about gut health often use simple language, but the underlying concepts depend on microorganisms, dietary substrates, dose, viability, food matrices, and health outcomes (9, 10). When these definitions are blurred, scientific terms can become broad marketing cues. Consumers may then struggle to judge whether food labels, product claims, and functional-food messages are based on evidence or simply on familiar health language.

In this context, objective nutrition knowledge refers to accurate factual knowledge (11). In the present study, it was operationalized through four separately presented questions covering the definitions and natural dietary sources of probiotics and prebiotics. Perceived awareness refers to self-assessed familiarity with a topic and was measured through participants' ratings of their familiarity with probiotics and prebiotics. Objective nutrition knowledge reflects factual accuracy, whereas perceived awareness reflects self-assessment. Accordingly, higher perceived awareness does not necessarily indicate greater objective knowledge (11, 12).

This distinction is important when interpreting previous population-based studies, which have assessed related but non-equivalent constructs. For example, among Australian adults, awareness of gut health, probiotics, and prebiotics was examined alongside attitudes and probiotic use (13). In Turkey, awareness and knowledge of probiotic dairy products varied across sociodemographic groups, with higher education, income, and female sex associated with greater awareness or purchase probability (14). Studies from China and India have further assessed knowledge, attitudes, and practices related to probiotics or probiotic foods in large adult samples (15, 16). However, these studies have not consistently distinguished self-perceived familiarity from objectively assessed knowledge.

Assessing knowledge of both definitions and natural dietary sources is therefore important. Definition-based items assess whether participants can correctly identify the two concepts, whereas source-based items assess whether they can recognize their occurrence in the diet (17). Including both probiotics and prebiotics is also warranted because familiarity with the two concepts may differ. Al-Maseimi et al. (18) reported lower familiarity with prebiotics than probiotics among Jordanian athletes. Information sources also warrant consideration. In a study of dietary supplements, Karbownik et al. (19) found that objective knowledge was associated with trust in advertising and the sources used to obtain information. Health beliefs are also relevant because the benefits attributed to probiotics and prebiotics depend on the specific microorganism or substrate, dose, and health outcome (10, 18–21).

Previous UAE research has provided an important foundation for this topic. Barqawi et al. (20) assessed knowledge, attitudes, and practices related to the microbiota and factors affecting its composition, including probiotic awareness and factual knowledge. Alqaydi et al. (21) broadened the local evidence by addressing both probiotics and prebiotics within a knowledge, attitudes, and practices framework. Building on this foundation, a more specific question remains: whether perceived awareness corresponds to objective nutrition knowledge for both probiotics and prebiotics, particularly when their definitions and natural dietary sources are assessed separately. A related question is whether commonly used information sources are independently associated with adequate objective knowledge.

The UAE provides a relevant setting for this study because of its demographic diversity, as the country is home to more than 200 nationalities (22). This demographic diversity is accompanied by varied nutrition-related patterns (23). In addition, active Internet users account for 99% of the population (24), making digital channels particularly relevant when examining where adults obtain information about probiotics and prebiotics (24–29).

This study therefore aimed to assess perceived awareness, objective nutrition knowledge, information sources, and health beliefs related to probiotics and prebiotics among adults in the UAE. It also examined adjusted associations of selected participant characteristics and information sources with adequate objective knowledge.

## 2 Materials and methods

### 2.1 Study design and participants

This cross-sectional study was conducted among adult residents in the United Arab Emirates from April 1 to May 30, 2026. Eligible participants were adults aged 18 years or older who were residing in the UAE at the time of data collection. Participants were recruited using online convenience sampling. The Google Forms survey link was distributed through WhatsApp and email to accessible contacts residing in different emirates of the UAE. Efforts were made to reach eligible adult residents across all seven emirates and to avoid recruitment from a single institutional network. A total of 744 complete responses were included in the final analysis.

The study was approved by the Research Ethics Unit at the University of Sharjah (approval no. REC-26–03-20-01-S). All participants provided electronic informed consent before starting the online questionnaire. No identifiable personal information was collected.

### 2.2 Questionnaire development and measures

The questionnaire was adapted from a previously published survey by Alkhaldy (17), which had itself been adapted from previously published surveys. For the present study, selected wording, response options, and response formats were adapted to improve clarity and contextual suitability for adults in the UAE, while retaining the main domains of the source questionnaire. The questionnaire was prepared in English and translated into Arabic using the Brislin back-translation method (25, 26). Before data collection, it was reviewed by three subject experts: one expert in food science and two experts in nutrition. The experts reviewed the relevance and clarity of the items, the questionnaire structure, ease of navigation, comprehensibility of the questions, and suitability of the response options. The questionnaire was refined based on their feedback. The final version was available in Arabic and English and required approximately 10 min to complete. The complete questionnaire is provided as Supplementary Questionnaire S1.

The questionnaire included four main parts. The first part collected sociodemographic and health-related information, including age, gender, educational level, work status, income, field of study, emirate of residence, doctor-diagnosed long-term health condition, smoking status, height, and weight. Body mass index (BMI) was calculated from self-reported weight in kilograms divided by height in meters squared (kg/m^2^) and categorized according to World Health Organization cutoffs (27).

The second part assessed perceived awareness of probiotics and prebiotics using a 10-point self-rated scale, where 1 indicated no awareness and 10 indicated the highest level of awareness. Scores were grouped as low awareness (1–3), moderate awareness (4–7), and high awareness (8–10). Participants were also asked to report the first word or phrase that came to mind when hearing the terms probiotics and prebiotics.

The third part assessed objective nutrition knowledge using all four questions addressing this construct in the questionnaire used by Alkhaldy (17). These questions covered the definitions and natural dietary sources of probiotics and prebiotics. All four questions were included because they provided a parallel assessment of factual knowledge across both concepts and aligned directly with the study objective. Their relevance and clarity were considered during the expert review described above. Correct answers were scored as 1, whereas incorrect, “don't know,” and “never heard” responses were scored as 0. The total score ranged from 0 to 4. Objective knowledge was classified as low (0–1 correct answers), moderate (2–3 correct answers), and high (4 correct answers). For regression analysis, adequate objective knowledge was defined as three or more correct answers out of four.

The fourth part assessed sources of dietary information and health beliefs related to probiotics and prebiotics. Participants reported their sources of dietary information from a predefined list and could select more than one source. They were also asked whether they perceived probiotics and prebiotics as beneficial, not beneficial, or did not know for selected health outcomes.

The questionnaire components used different response formats and were not designed to form homogeneous multi-item scales. The four-item objective nutrition knowledge score was treated as a criterion-referenced index. Content validity was addressed by basing the index on a published questionnaire, aligning its items with the study objectives, and obtaining expert review of item relevance and clarity. Internal consistency was therefore not assessed. Test–retest reliability was not evaluated.

### 2.3 Sample size calculation

The minimum sample size was estimated using Raosoft software based on the target adult population in the United Arab Emirates (28). Assuming a 95% confidence level, a 5% margin of error, and a 50% response distribution, the minimum required sample size was 385 participants. The final sample exceeded this minimum and included 744 participants.

### 2.4 Statistical analysis

Data were analyzed using IBM SPSS Statistics version 31 (IBM Corp., Armonk, NY, USA) and Microsoft Excel. Categorical variables were presented as frequencies and percentages. Perceived awareness and objective knowledge scores were summarized using the mean ± standard deviation and median with interquartile range. Statistical significance was set at *p* < 0.05. Open-ended responses were manually reviewed; blank, unclear, unrelated, and “don't know” responses were excluded. Similar words or phrases were standardized and grouped before generating the word cloud.

#### 2.4.1 Multivariable logistic regression

Multivariable logistic regression was used to examine adjusted associations with adequate objective knowledge, defined as ≥3 correct answers out of 4. The model included age, gender, educational level, field of study, employment status, monthly income, emirate, long-term health condition, and three focal information sources: Internet/websites, social media, and healthcare professionals. All variables were entered simultaneously.

The three information sources were selected because they represent conceptually distinct pathways of nutrition-information acquisition: self-directed digital access, socially mediated exposure, and professional health guidance. Marital status was not included because its expected relationship with objective knowledge was considered indirect relative to the educational, socioeconomic, and information-source variables included in the model. BMI and smoking status were retained for descriptive characterization but were outside the primary analytic focus. Perceived awareness and health beliefs were treated as parallel study constructs and were therefore not entered as covariates. Adjusted odds ratios (aOR) and 95% confidence intervals were reported. The complete model is presented in Supplementary Table S1.

## 3 Results

### 3.1 Participant characteristics

A total of 744 adult residents in the UAE completed the survey. Participants varied by age, gender, educational level, employment status, income, field of study, emirate of residence, and health-related characteristics, as shown in Table 1. Based on BMI classification, 45.8% of participants were overweight and 14.9% were obese (Table 1).

**Table 1:** Characteristics of the study participants (*N* = 744).

Characteristic	*n* (%)
Age (years)	
18–24	295 (39.7)
25–39	195 (26.2)
40–59	212 (28.5)
≥60	42 (5.6)
Gender	
Male	389 (52.3)
Female	355 (47.7)
Marital status	
Single	340 (45.7)
Married	308 (41.4)
Divorced	75 (10.1)
Widowed	21 (2.8)
Educational level	
Less than high school	18 (2.4)
High school or equivalent	189 (25.4)
Bachelor's degree	492 (66.1)
Master's degree or higher	45 (6.0)
Work status	
Student	267 (35.9)
Employed	338 (45.4)
Unemployed	57 (7.7)
Retired	33 (4.4)
Business/trading	49 (6.6)
Income, AED per month	
No income	224 (30.1)
Below 5,000 AED	61 (8.2)
5,000 to 10,000 AED	45 (6.0)
10,001 to 15,000 AED	56 (7.5)
15,001 to 20,000 AED	156 (21.0)
20,001 AED or more	202 (27.2)
Field of study	
Medicine or medical sciences	190 (25.5)
Science	149 (20.0)
Literature	325 (43.7)
No specific field	80 (10.8)
Emirate of residence	
Abu Dhabi	245 (32.9)
Dubai	115 (15.5)
Sharjah	173 (23.3)
Ajman	52 (7.0)
Umm Al Quwain	26 (3.5)
Ras Al Khaimah	93 (12.5)
Fujairah	40 (5.4)
Doctor-diagnosed long-term health condition	
Yes	224 (30.1)
No	431 (57.9)
Not sure	89 (12.0)
Smoking status	
No	485 (65.2)
Yes	188 (25.3)
Ex-smoker	71 (9.5)
BMI category, kg/m^2a^	
Underweight	41 (5.5)
Normal weight	251 (33.7)
Overweight	341 (45.8)
Obese	111 (14.9)

Values are presented as *n* (%). Percentages were calculated using *N* = 744 and rounded to one decimal place.

^a^BMI was calculated from self-reported height and weight.

### 3.2 Perceived awareness and objective knowledge

Table 2 summarizes perceived awareness and objective knowledge of probiotics and prebiotics. Perceived awareness was modest (4.6 ± 2.8), and only 18.0% of participants reported high awareness. Objective knowledge was also limited, with a mean score of 1.9 ± 1.6 out of 4. Overall, 45.2% of participants had low objective knowledge. Knowledge was weaker for prebiotics than probiotics, particularly for identifying natural prebiotic sources (37.0%).

**Table 2:** Perceived awareness and objective knowledge of probiotics and prebiotics among participants (*N* = 744).

Domain	Score, mean ±*SD*	Score, median (IQR)	Category/item	*n* (%)
Perceived awareness	4.6 ± 2.8	4 (2–7)	Low awareness	305 (41.0)
			Moderate awareness	305 (41.0)
			High awareness	134 (18.0)
Objective knowledge score	1.9 ± 1.6	2 (0–4)	Correct probiotic definition	415 (55.8)
			Correct prebiotic definition	311 (41.8)
			Correct probiotic source	393 (52.8)
			Correct prebiotic source	275 (37.0)
Objective knowledge level	–	–	Low objective knowledge	336 (45.2)
			Moderate objective knowledge	213 (28.6)
			High objective knowledge	195 (26.2)

Values are presented as *n* (%) unless otherwise indicated. Perceived awareness was assessed using a 10-point self-rated scale and classified as low, moderate, and high using scores 1–3, 4–7, and 8–10, respectively.

Objective knowledge was assessed using four items, with a total score ranging from 0 to 4. Low, moderate, and high objective knowledge were defined as 0–1, 2–3, and 4 correct answers, respectively.

SD, standard deviation; IQR, interquartile range.

Open-ended responses to the first word or phrase associated with probiotics and prebiotics are presented descriptively as a word cloud in Supplementary Figure S1. Among informative open-text responses (*n* = 483), the most frequent standardized terms were bacteria (*n* = 160), health (*n* = 139), beneficial (*n* = 119), gut (*n* = 116), food (*n* = 74), yogurt (*n* = 69), and digestion (*n* = 46).

### 3.3 Perceived awareness in relation to objective knowledge

**Figure 1:**
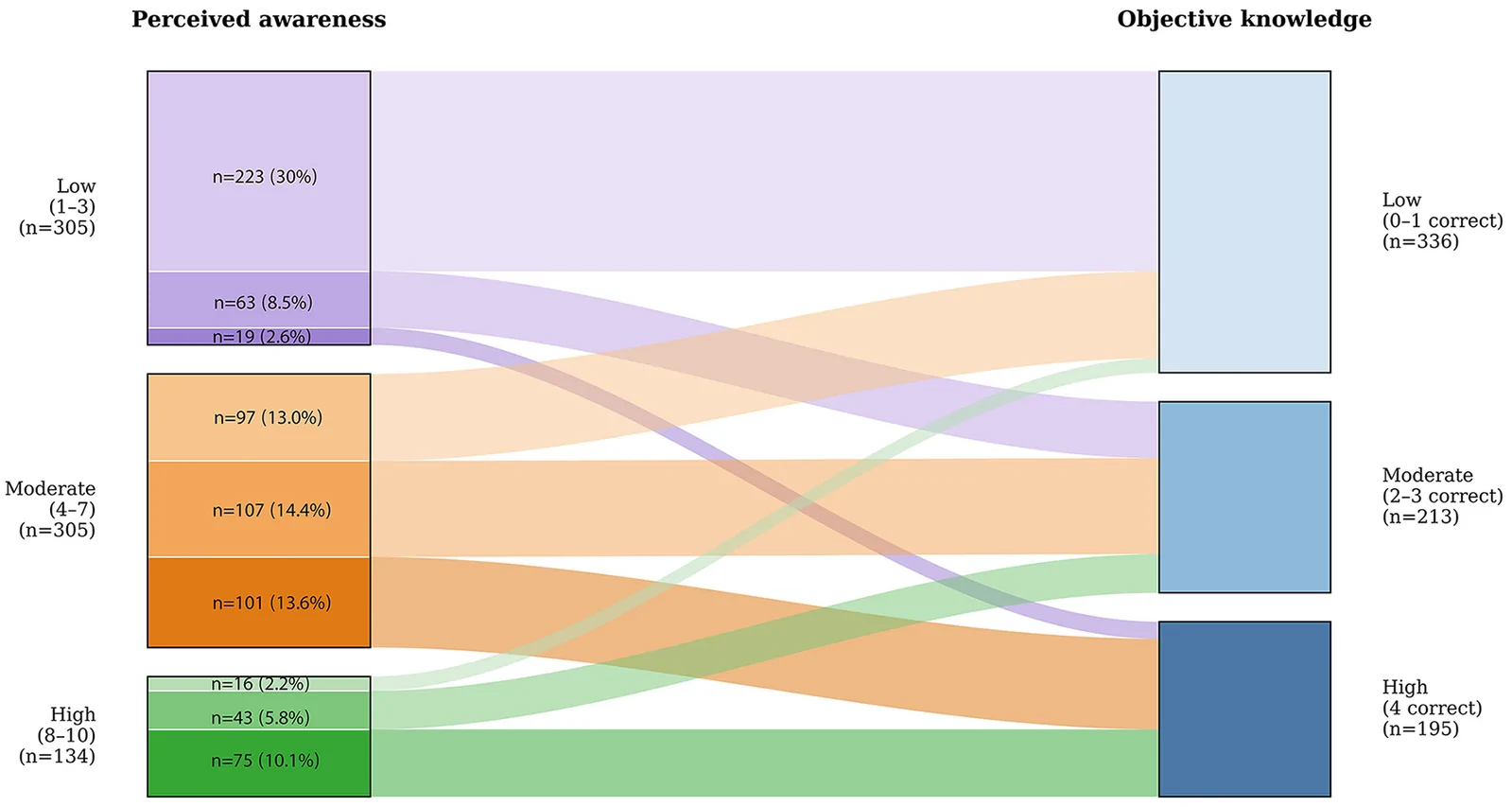
Perceived awareness vs. objective knowledge of probiotics and prebiotics among adults in the United Arab Emirates. Perceived awareness was assessed using a 10-point self-rated scale and classified as low (1–3), moderate (4–7), and high (8–10). Objective knowledge was assessed using four items and classified as low (0–1 correct answer), moderate (2–3 correct answers), and high (4 correct answers). Flow widths represent the number of participants in each perceived-awareness and objective-knowledge combination. Values shown in the figure are *n* (%), with percentages calculated from the total sample (*N* = 744).

Figure 1 shows the distribution of objective knowledge across perceived awareness levels. The largest subgroup had both low perceived awareness and low objective knowledge (30.0%). The moderate-awareness group was distributed across all objective knowledge levels. Only 10.1% of the total sample had both high perceived awareness and high objective knowledge.

### 3.4 Information sources and health beliefs

**Figure 2:**
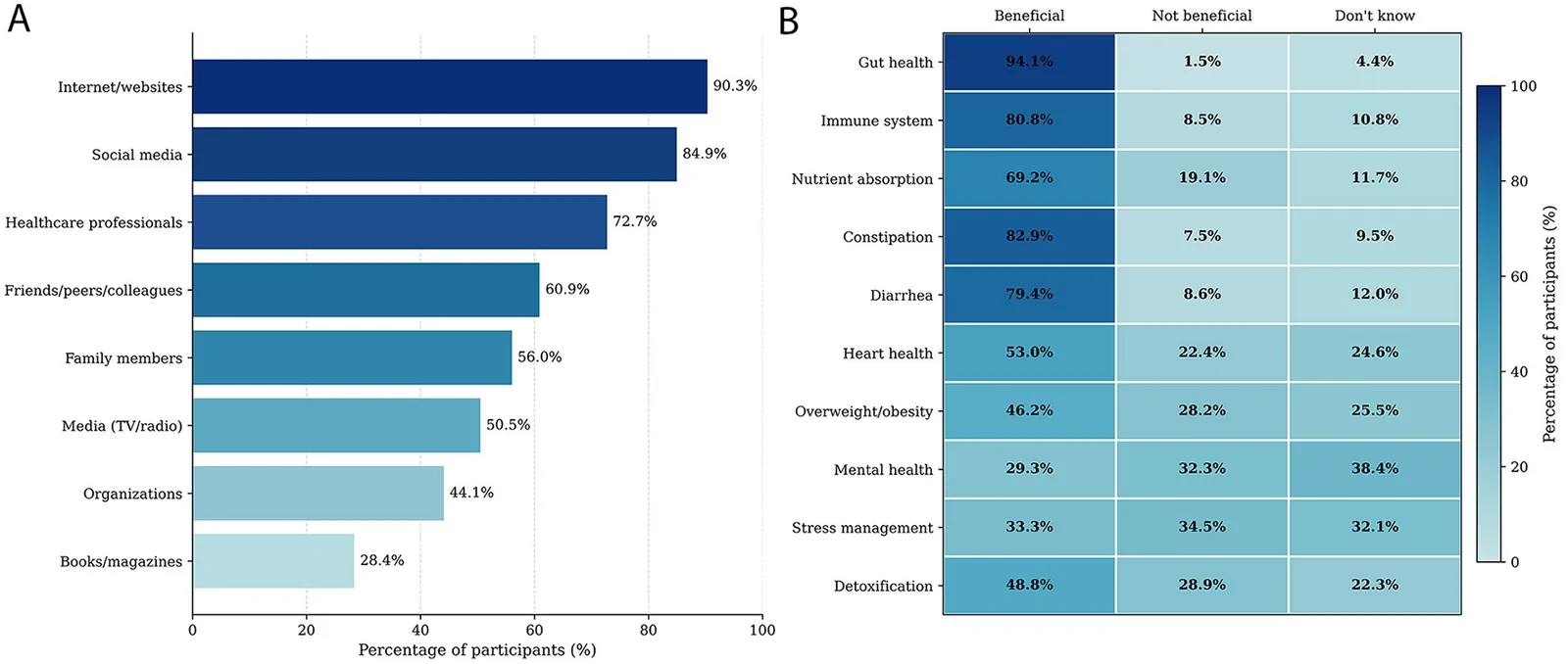
Information sources and health beliefs related to probiotics and prebiotics among adults in the United Arab Emirates. **(A)** Sources of dietary information. Participants could select more than one source (*N* = 744). **(B)** Beliefs regarding the potential beneficial effects of probiotics and prebiotics. Values are row percentages calculated from *N* = 744 participants across three response categories: beneficial, not beneficial, and don't know. Darker shading indicates a higher percentage of participants.

Figure 2 summarizes information sources and perceived health benefits. Internet/websites (90.3%) and social media (84.9%) were the most commonly reported sources, followed by healthcare professionals (72.7%). Perceived benefits were highest for gut health (94.1%), constipation (82.9%), immunity (80.8%), and diarrhea (79.4%). Responses were more uncertain for detoxification, overweight or obesity, mental health, and stress management.

### 3.5 Factors associated with adequate objective knowledge

**Figure 3:**
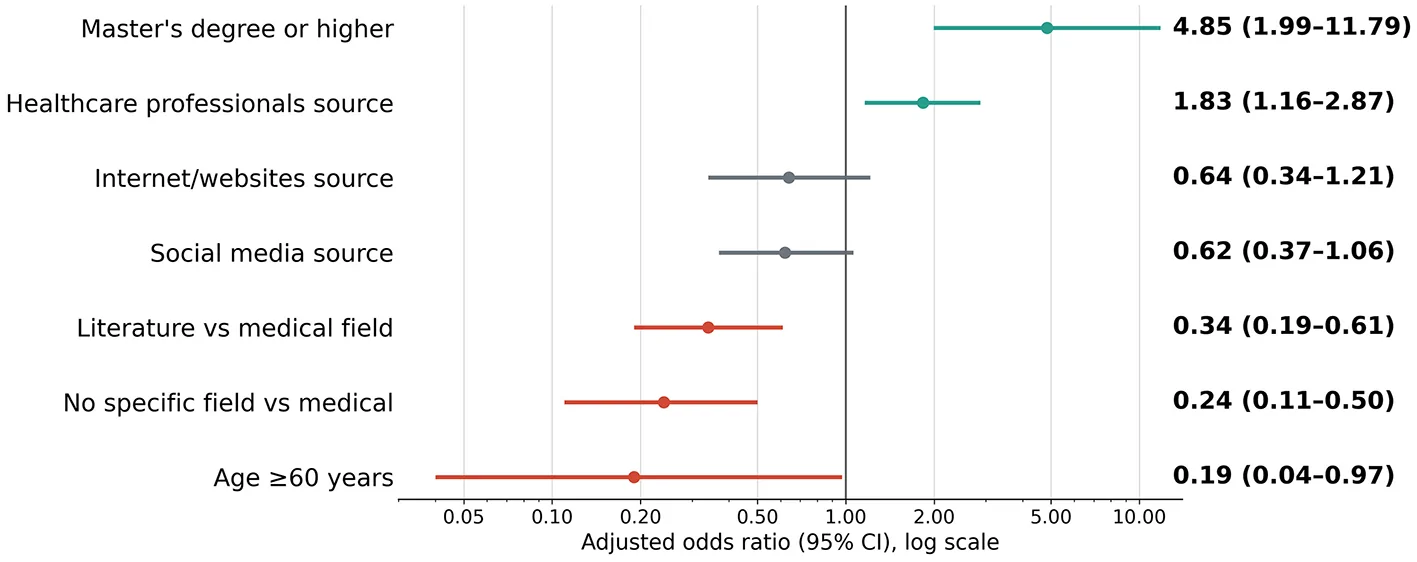
Selected adjusted associations of participant characteristics and information sources with adequate objective knowledge of probiotics and prebiotics. Adjusted odds ratios and 95% confidence intervals were derived from the multivariable logistic regression model. The vertical line indicates an adjusted odds ratio of 1.0. Adequate objective knowledge was defined as ≥3 correct answers out of 4. The full regression model is provided in Supplementary Table S1.

Figure 3 presents selected adjusted associations with adequate objective knowledge, while the full multivariable logistic regression model is provided in Supplementary Table S1. Overall, 39.9% of participants had adequate objective knowledge. In the multivariable model, adequate knowledge was higher among participants with a master's degree or higher and among those who used healthcare professionals as an information source. Lower odds were observed among participants aged ≥60 years and among those from literature or no specific field compared with medicine or medical sciences. Internet/websites and social media were not independently associated with adequate objective knowledge.

## 4 Discussion

This study examined perceived awareness, objective nutrition knowledge, information sources, and health beliefs about probiotics and prebiotics among adults in the UAE. The main finding was a mismatch between familiarity and accurate understanding. Participants were widely exposed to nutrition information, especially through digital sources. Despite this exposure, objective nutrition knowledge remained limited and was weakest for prebiotics and their dietary sources. This pattern indicates a nutrition-related knowledge gap in which exposure to probiotic and prebiotic terms does not necessarily translate into accurate understanding. Health beliefs were strongest for gut-related outcomes and immunity and more uncertain for detoxification, obesity, stress, and mental health.

The knowledge gaps observed are important because most participants had university-level education. This suggests that limited understanding of probiotics and prebiotics is not confined to low educational attainment. Previous UAE studies have reported that awareness of probiotics may coexist with incomplete knowledge (20, 21). In the present sample, 45.8% of participants were overweight and 14.9% were obese, yielding a combined prevalence of 60.7%. This was lower than the national prevalence of overweight and obesity (BMI ≥ 25 kg/m^2^; 67.9%) and obesity alone (27.8%) reported in the UAE National Health Survey 2017–2018 (29). However, it was broadly comparable with a population-based survey in Dubai, which reported 39.8% overweight and 17.8% obesity (30). Differences in sampling and the use of self-reported height and weight may partly explain these variations. The high prevalence of overweight and obesity highlights the need for accurate communication. Claims linking probiotics and prebiotics to weight management should reflect the strength of the available evidence. More broadly, scientifically supported concepts should be distinguished from general wellness messaging. This is particularly important in digital environments, where nutrition information varies in quality and accuracy (31).

The modest awareness score and low objective knowledge score clarify the nature of the gap. Many participants appeared to have partial and uneven knowledge, especially for prebiotics and their dietary sources. Probiotics may therefore be more recognizable to the public, while prebiotics remain less clearly understood. Findings from Türkiye are in line with this pattern, with higher reported knowledge of probiotics than prebiotics among adult consumers (87.0% vs. 62.2%) (32). A similar imbalance was reported among Saudi adults, where correct responses were lower for prebiotic than probiotic definitions and food sources (17). Compared with that study, the present sample showed lower correct responses across the four core knowledge items, with the largest difference observed for natural prebiotic sources (37.0% vs. 68.8%). Prebiotic-focused research in Romania also showed a gap between familiarity and understanding; 74% were familiar with the term, but only 40% reported understanding it (33). For nutrition education, this distinction is important. General messages about “gut health” may increase familiarity. However, they may not build the conceptual knowledge needed to interpret food examples and product claims. Educational strategies should therefore explain the difference between probiotics and prebiotics. They should also link each concept to appropriate dietary sources. Usable nutrition information depends on exposure, clarity, trust, and relevance to everyday food contexts (34).

The distribution of perceived awareness against objective knowledge further shows that self-rated awareness should not be treated as a proxy for nutrition literacy. The largest subgroup had both low perceived awareness and low objective knowledge. Only 10.1% of the total sample combined high awareness with high objective knowledge. The moderate-awareness group was spread across all knowledge levels, suggesting that familiarity with the terms does not consistently indicate accurate understanding. This agrees with broader nutrition-literacy evidence showing that self-perceived nutrition knowledge can differ from objectively measured knowledge (12). Recognition of probiotics and prebiotics does not necessarily mean that adults can define them correctly or identify their dietary sources. The supplementary word cloud (Supplementary Figure S1) supports this interpretation. Frequent first-association terms included broad words such as health and beneficial, alongside gut- and food-related terms such as bacteria, gut, yogurt, food, and digestion. This pattern reflects familiarity with common probiotic- and prebiotic-related language, but not necessarily precise knowledge of definitions or dietary sources. Awareness campaigns should therefore be paired with simple conceptual messages that correct basic misunderstandings and connect each term to appropriate food examples.

The pattern of information sources adds an important nutrition communication dimension to the findings. Internet websites and social media were the dominant sources of dietary information. Neither source was independently associated with adequate objective knowledge after adjustment (*p* = 0.173 and *p* = 0.080, respectively). By contrast, healthcare professionals were positively associated with adequate knowledge (*p* = 0.009). This contrast suggests that access to nutrition messages is not enough; the source and interpretability of the information also matter. From a health-literacy perspective, this is not only an individual knowledge gap; health information also needs to be trustworthy, understandable, and actionable (35). Digital platforms can increase exposure to probiotics and prebiotics, but their messages may be simplified, commercial, or inconsistent. In nutrition, confident digital claims can make weak evidence appear credible (36). Trust in nutrition information sources is linked with how people seek and use dietary information. Scientifically supported communication should therefore be present in the digital spaces adults already use. Alongside this, professional guidance should be strengthened to support accurate and practical understanding (37). This supports a source-sensitive communication approach in which healthcare professionals help translate microbiome-related terms into accurate and practical dietary guidance. Digital messages should also be improved for clarity and evidence quality, especially in online spaces where health-related misinformation can spread widely (38).

The stronger endorsement of gut health, constipation, diarrhea, and immunity is biologically plausible. These outcomes are commonly discussed in relation to gut-mediated effects and host-microbiota interactions (10). Responses were more uncertain for detoxification, obesity, stress, and mental health. Popular microbiome communication often presents gut health as an actionable wellness trend, while giving less attention to the underlying science. Such uncertainty is important because these outcomes sit closer to the boundary between established benefits, emerging evidence, and broad wellness claims (39). Public-facing nutrition messages should avoid presenting probiotics and prebiotics as general health-promoting products. Clinical guidance similarly stresses that effects are condition-, strain-, substance-, and dose-specific, and should not be treated as universal (40). Messages should clarify that potential effects depend on the specific probiotic strain, prebiotic compound, food source, dose, and health outcome.

The factors associated with adequate objective knowledge, summarized in Figure 3 and Supplementary Table S1, suggest that nutrition literacy was unevenly distributed across participants. Higher education was important, particularly for participants with a master's degree or higher [adjusted odds ratio (aOR) 4.85, *p* < 0.001]. When field of study was examined, participants from literature or no specific field had lower odds of adequate knowledge than those from medicine or medical sciences (*p* < 0.001 for both). This pattern suggests that general education alone may be insufficient when the topic requires understanding of microorganisms, dietary fibers, and food sources. Recent evidence from China similarly supports the role of specialized exposure, as medical-related employment was independently associated with good probiotic knowledge (15). Still, specialized background does not guarantee full understanding. In an international survey, health professionals' probiotic knowledge varied by profession, and only 8.9% rated it as excellent (41). The lower odds among adults aged 60 years or older (aOR 0.19, *p* = 0.045) may reflect differences in exposure to nutrition terminology, digital information, or structured nutrition education. For older adults, this is relevant because nutrition information can be difficult to interpret without accessible and usable learning tools (42).

Supplementary Table S1 showed statistical significance for some categories. These included unemployment (*p* = 0.047), income of 15,001–20,000 UAE dirhams (AED) (*p* = 0.002), and residence in Dubai (*p* = 0.031). However, these variables should not be interpreted as direct causes of knowledge. They may reflect broader differences in education, access to health services, digital exposure, or trusted information sources. Previous studies support this interpretation, linking health literacy to socioeconomic conditions (43, 44). Accordingly, income, work status, and emirate of residence are better viewed as markers of information environments.

### 4.1 Strengths and limitations

By including 744 adult residents from all seven emirates, this study provides UAE-specific evidence on nutrition-related knowledge and functional food literacy related to probiotics and prebiotics. A key strength is the distinction between perceived awareness and objective knowledge. This allowed the study to show that familiarity with these terms does not necessarily reflect accurate understanding of definitions or dietary sources. The study also examined information sources and health beliefs in the same sample. This is important because public understanding of probiotics and prebiotics may be influenced by both the information people receive and the health claims they associate with these terms.

However, the use of non-probability online convenience sampling may have introduced selection bias because participants were recruited through accessible online networks. Although efforts were made to reach adult residents across all seven emirates, the findings should not be interpreted as nationally representative of all adult residents in the UAE. Online recruitment may also have favored digitally connected adults. Objective knowledge was assessed using four core items that captured basic definitions and dietary-source knowledge. The measure was intentionally brief to reduce respondent burden and survey fatigue in a general-population online survey. Because it focused on core knowledge only, it may not capture the full range of nutrition literacy related to probiotics and prebiotics. Perceived awareness and health beliefs were assessed using the combined term “pro/prebiotics”. This wording may have limited our ability to determine whether all participants consistently distinguished between the two concepts. BMI, information sources, and health beliefs were self-reported. The study identified which sources participants used, but did not assess the quality, frequency, or accuracy of the specific information they accessed. It also did not measure actual use of probiotic or prebiotic foods or supplements, behavioral intention, or food choice. Because of the cross-sectional design, the findings show associations only and cannot establish causality. Future studies should use probability-based sampling, broader nutrition-literacy tools, dietary-use measures, and direct assessment of digital information quality.

### 4.2 Conclusion

This study examined perceived awareness, objective nutrition knowledge, health beliefs, and information sources related to probiotics and prebiotics among adults in the UAE. The findings show that perceived awareness did not consistently correspond to objective knowledge. This mismatch indicates a nutrition-related knowledge gap, especially for prebiotics and their dietary sources. The study also identifies a specific communication challenge in a highly connected setting. Digital sources were widely used, but this exposure was not independently associated with adequate knowledge. In contrast, healthcare professionals were more clearly linked with accurate understanding. Nutrition communication should therefore move beyond increasing familiarity with probiotics and prebiotics and should make these concepts easier to interpret. Messages should clearly distinguish probiotics from prebiotics. They should link each concept to appropriate dietary sources and address common health beliefs. At the same time, probiotics and prebiotics should not be presented as general wellness solutions. In the UAE, evidence-based nutrition education and public health communication strategies are needed. These efforts should strengthen professional guidance, improve digital nutrition messages, and help adults evaluate microbiome-related food and health claims more accurately.

## Data Availability

The raw data supporting the conclusions of this article will be made available by the authors, without undue reservation.
