# Supplementary material for: Perceived awareness, objective nutrition knowledge, and health beliefs about probiotics and prebiotics among adults in the United Arab Emirates: a cross-sectional study
Source: Front Nutr. 2026 Sep 7;13:1929193. doi: 10.3389/fnut.2026.1929193 (PMC13590363; doi:10.3389/fnut.2026.1929193)
Supplement: Supplementary file 1 [file Table_1.docx]

**Supplementary Material**

Supplementary Questionnaire S1. Questionnaire used to collect sociodemographic and health-related information and assess perceived awareness, objective nutrition knowledge, information sources, and health beliefs related to probiotics and prebiotics

| **Section 1: Sociodemographic and background section:** | |
| --- | --- |
| 1. How old are you?  - 18–24 years - 25–39 years - 40–59 years - ≥60 years | 2. Gender:  - Male - Female |
| 3. What is your marital status?  - Single - Married - Divorced - Widowed | 4. What is your highest level of education?  - Less than high school - High school or equivalent - Bachelor's degree - Master’s degree or higher |
| 5. What is your current work status?  - Student - Employed - Unemployed - Retired - Business/trading | 6. What is your monthly income range? (AED(  - No income - Below 5,000 AED - 5,000 to 10,000 AED - 10,001 to 15,000 AED - 15,001 to 20,000 AED - 20,001 AED or more |
| 7. What is your specialty?  - Medicine or medical sciences - Science - Literature - No specific field (high school students/education was not completed) | 8. Have you ever been diagnosed by a doctor with a long-term health condition (lasting 3 months or more)?  - Yes, I have been diagnosed - No, I haven't been diagnosed - Not sure |
| 9. Do you smoke?  - Yes - No - I am an ex-smoker | 10. What is your height?   ……………. cm |
| 11. How much do you weigh?   ……………. kg | 12. From where do you seek information about your diet? Select all that apply  - Family members - Friends/peers/colleagues - Books/Magazines - Internet website - Media (TV, radio) - Social media (Facebook, Twitter, Instagram, etc) - Health care professionals (doctor, nurse, dietitian, etc) - Organizations (MOH, WHO, government, associations) |
| 13. In which emirate do you currently reside?  - Abu Dhabi - Dubai - Sharjah - Ajman - Umm Al Quwain - Ras Al Khaimah - Fujairah | |
| **Section 2: Questions related to awareness** | |
| 1. Are you aware of the concept of pro/prebiotics? (1 means you do not know anything about it, and 10 means you know a great deal of information) **1** \| **2** \| **3** \| **4** \| **5** \| **6** \| **7** \| **8** \| **9** \| **10** | 2. What was the first thing that came into your mind when hearing pro/prebiotics?   ………………….. |
| **Section 3: Questions related to knowledge** | |
| 1. To the best of your knowledge, what are **’probiotics’**?  - Live microorganisms that when digested can be beneficial to humans - A vitamin supplement - Antibiotics prescribed to treat infections caused by bacteria - Don’t know - Never heard | 2. To the best of your knowledge, what are **’prebiotics’**?  - Dietary fiber that good bacteria need to survive - A vitamin supplement - Antibiotics prescribed to treat infections caused by bacteria - Don’t know - Never heard |
| 3. To the best of your knowledge, which one of the following foods may be a natural source of **probiotics**?  - Yoghurt - Fruit and vegetables and whole grains - Foods cannot be a natural source of probiotics - Don’t know - Never heard | |
| 4. To the best of your knowledge, which one of the following foods may be a natural source of **prebiotics**?  - Yoghurt - Fruit and vegetables and whole grains - Foods cannot be a natural source of prebiotics - Don’t know - Never heard | |
| **Section 4: Questions related to health beliefs** | |
| 1. Do you believe that pro/prebiotics is beneficial for: \| **Pro/prebiotics** \| \|; \| Beneficial \| Not beneficial \| I do not know; **Overall digestion/gut health** \| \| \|; **Support the immune system** \| \| \|; **Absorption of nutrients** \| \| \|; **Detoxify the body** \| \| \|; **Stress management** \| \| \|; **Constipation** \| \| \|; **Diarrhea** \| \| \|; **Heart health** \| \| \|; **Overweight/obesity** \| \| \|; **Mental health** \| \| \| | |

Thank you for your time and participation

Supplementary Figure S1. Word cloud of open-ended responses to the first word or phrase associated with probiotics and prebiotics. Larger words indicate more frequently mentioned standardized terms. Arabic responses were translated into English before generating the word cloud. Blank and non-informative responses were excluded from term-frequency counts, and a response could contribute to more than one standardized term.

Supplementary Table S1. Adjusted associations of selected participant characteristics and information sources with adequate objective knowledge of probiotics and prebiotics (N = 744).

| **Characteristic** | **Adequate objective knowledge n/N (%)** | **Adjusted odds ratio (95% CI)** | ***p*-value** |
| --- | --- | --- | --- |
| **Age group** | |  | |
| 18-24 years | 189/295 (64.1) | Ref. |  |
| 25-39 years | 65/195 (33.3) | 0.84 (0.34-2.06) | 0.701 |
| 40-59 years | 37/212 (17.5) | 0.47 (0.17-1.25) | 0.129 |
| ≥60 years | 6/42 (14.3) | 0.19 (0.04-0.97) | 0.045 |
| **Gender** | | | |
| Female | 213/355 (60.0) | Ref. |  |
| Male | 84/389 (21.6) | 0.69 (0.38-1.22) | 0.200 |
| **Educational level** | | | |
| High school or lower | 94/207 (45.4) | Ref. |  |
| Bachelor's degree | 175/492 (35.6) | 1.26 (0.79-2.01) | 0.329 |
| Master’s degree or higher | 28/45 (62.2) | 4.85 (1.99-11.79) | <0.001 |
| **Field of study** | | | |
| Medicine or medical sciences | 138/190 (72.6) | Ref. |  |
| Science | 69/149 (46.3) | 0.64 (0.36-1.12) | 0.115 |
| Literature | 73/325 (22.5) | 0.34 (0.19-0.61) | <0.001 |
| No specific field | 17/80 (21.2) | 0.24 (0.11-0.50) | <0.001 |
| **Work status** | | | |
| Employed | 73/338 (21.6) | Ref. |  |
| Student | 174/267 (65.2) | 1.76 (0.66-4.71) | 0.257 |
| Unemployed | 31/57 (54.4) | 2.50 (1.01-6.18) | 0.047 |
| Retired | 5/33 (15.2) | 1.64 (0.34-8.03) | 0.539 |
| Business/trading | 14/49 (28.6) | 1.60 (0.73-3.52) | 0.239 |
| **Monthly income, AED** | | | |
| 20,001 AED or more | 58/202 (28.7) | Ref. |  |
| No income | 143/224 (63.8) | 0.69 (0.27-1.73) | 0.426 |
| Below 5,000 AED | 38/61 (62.3) | 0.85 (0.32-2.31) | 0.756 |
| 5,000 to 10,000 AED | 24/45 (53.3) | 0.82 (0.32-2.09) | 0.677 |
| 10,001 to 15,000 AED | 12/56 (21.4) | 0.55 (0.22-1.36) | 0.193 |
| 15,001 to 20,000 AED | 22/156 (14.1) | 0.38 (0.20-0.71) | 0.002 |
| **Emirate of residence** | | | |
| Abu Dhabi | 65/245 (26.5) | Ref. |  |
| Dubai | 68/115 (59.1) | 1.92 (1.06-3.46) | 0.031 |
| Sharjah | 110/173 (63.6) | 1.18 (0.63-2.20) | 0.599 |
| Ajman | 19/52 (36.5) | 0.83 (0.36-1.91) | 0.664 |
| Umm Al Quwain | 7/26 (26.9) | 1.27 (0.44-3.62) | 0.660 |
| Ras Al Khaimah | 18/93 (19.4) | 0.86 (0.44-1.69) | 0.657 |
| Fujairah | 10/40 (25.0) | 0.61 (0.25-1.51) | 0.285 |
| **Doctor-diagnosed long-term health condition** | | | |
| No, I haven't been diagnosed | 192/431 (44.5) | Ref. |  |
| Yes, I have been diagnosed | 80/224 (35.7) | 1.12 (0.71-1.77) | 0.615 |
| Not sure | 25/89 (28.1) | 0.92 (0.49-1.69) | 0.778 |
| **Internet/websites as information source** | | | |
| No | 37/72 (51.4) | Ref. |  |
| Yes | 260/672 (38.7) | 0.64 (0.34-1.21) | 0.173 |
| **Social media as information source** | | | |
| No | 59/112 (52.7) | Ref. |  |
| Yes | 238/632 (37.7) | 0.62 (0.37-1.06) | 0.080 |
| **Healthcare professionals as information source** | | | |
| No | 48/203 (23.6) | Ref. |  |
| Yes | 249/541 (46.0) | 1.83 (1.16-2.87) | 0.009 |
| Adequate objective knowledge was defined as ≥3 correct answers out of 4; overall, 297/744 participants (39.9%) had adequate objective knowledge. Percentages in the “adequate objective knowledge” column were calculated within each category. AED: United Arab Emirates dirham | | | |
